# A Case of Postoperative Noncardiogenic Edema: Negative Pressure Pulmonary Edema

**DOI:** 10.5334/jbsr.3551

**Published:** 2024-07-03

**Authors:** Miloud Dewilde, Bart Lutin

**Affiliations:** 1 UZ Leuven, Leuven, Belgium; 2 AZ Groeninge, Kortrijk, Belgium

**Keywords:** Negative pressure pulmonary edema, postoperative non-cardiogenic edema, centrilobular ground glass opacities, chest CT, chest X-ray

## Abstract

A case of complete recovery of negative pressure pulmonary edema after a Cottle surgery in a 24-year-old male.

*Teaching point:* Negative pressure pulmonary edema is an important cause of postoperative noncardiogenic edema, with the spontaneous disappearance of all complaints within a relatively short period.

## Introduction

Negative pressure pulmonary edema (NPPE) is an important cause of postoperative noncardiogenic edema [1]. NPPE can result from laryngospasm or other types of upper airway obstruction following extubation [2, 3]. The etiology is multifactorial, but the main reported cause of NPPE is increased negative intrathoracic pressure with increased blood flow to the right heart. The pulmonary vascular bed dilates, the interstitial pressure around the capillaries decreases, with intravascular fluid is drawn into the interstitial space [4]. This worsens gas exchange and triggers a cascade of hypoxemia, catecholamine release, and systemic and pulmonary hypertension. The result is an acute increase in afterload, which worsens transcapillary fluid efflux and increases interstitial and alveolar edema [2].

## Case Report

**Figure 1:**
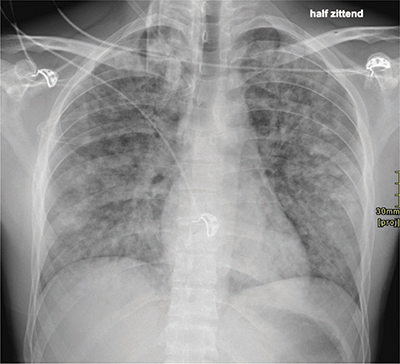
Chest X-ray at day 1.

A 24-year-old man with known Gilbert’s syndrome underwent a Cottle septorhinoplastia because of a post-traumatic external and internal deviation of the nose and the nasal septum. Two months earlier, the patient traveled through Costa Rica and Mexico and tested positive for SARS-CoV-II. The patient was currently asymptomatic and fully recovered. The surgery was performed under general anesthesia. Postoperatively, oxygen demand with tachycardia persisted without further tachypnea or distress. A chest X-ray (Figure 1) showed distributed flocculent opacificaties in both lungs. There was no apparent pleural effusion. The image could be consistent with an alveolar edema.

**Figure 2:**
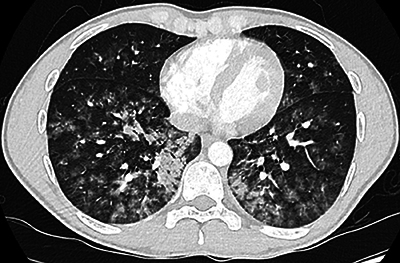
Chest CT at day 1.

A chest computed tomography (CT) examination was performed to exclude a possible pulmonary embolism. The chest CT (Figure 2) showed bilateral and diffusely spread ground glass opacities with a flocculent aspect. Some limited flocculent consolidation components were also observed in both lower lobes. Bilateral, diffusely distributed centrilobular nodules were present (tree-in-bud). There was no pleural effusion, and pulmonary embolism was excluded.

The differential diagnosis included both post-obstructive pulmonary edema (NPPE) and alveolar edema on medication or inhaled gases. Aspiration pneumonitis was considered unlikely.

**Figure 3:**
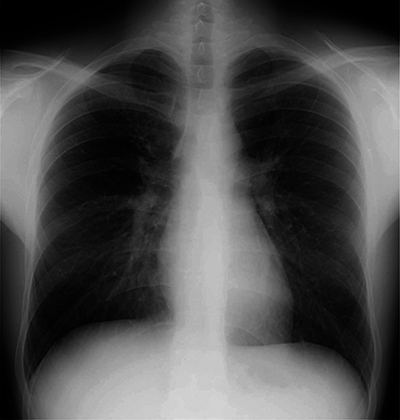
Chest X-ray at day 5.

After 5 days, a chest X-ray (Figure 3) showed complete regression of the pulmonary infiltrations, and the patient was discharged in good general condition without additional support.

## Discussion

It has been reported that NPPE occurs in less than 1/1000 surgical patients. Around 0.05% till 0.1% of all procedures involving intubation and general anesthesia may show NPPE, but it is often attributable to other etiologies [2, 3].

There are multiple risk factors, including obesity, a short neck, obstructive sleep apnea, or acromegaly. In addition, the risk of NPPE is considered to be higher after oral and maxillofacial surgery as compared to other types of surgery [5].

In cases of NPPE, supportive treatment is required. The patients receive supplemental oxygen, and hypervolemic patients may benefit from diuretics. In selected patients, bronchodilators and/or noninvasive continuous positive airway pressure (CPAP) may be helpful. Some patients will require reintubation [4]. In the long term, all complaints will disappear spontaneously within a relatively short period of time and without sequelae.

## Conclusion

NPPE is a significant postoperative complication, primarily provoked by increased negative intrathoracic pressure following upper airway obstruction, often associated with laryngospasm. Typically, there is a need for supportive management and, in some cases, necessity of reintubation. Most patients experience complete resolution of symptoms within a relatively short period and without long-term sequelae.
